# Cryo–electron microscopy structure of the H3-H4 octasome: A nucleosome-like particle without histones H2A and H2B

**DOI:** 10.1073/pnas.2206542119

**Published:** 2022-11-02

**Authors:** Kayo Nozawa, Yoshimasa Takizawa, Leonidas Pierrakeas, Chizuru Sogawa-Fujiwara, Kazumi Saikusa, Satoko Akashi, Ed Luk, Hitoshi Kurumizaka

**Affiliations:** ^a^Laboratory of Chromatin Structure and Function, Institute for Quantitative Biosciences, The University of Tokyo, Tokyo 113-0032, Japan;; ^b^School of Life Science and Technology, Tokyo Institute of Technology, Yokohama, Kanagawa 226-8501, Japan;; ^c^Department of Biochemistry and Cell Biology, Stony Brook University, Stony Brook, NY 11794;; ^d^National Metrology Institute of Japan, National Institute of Advanced Industrial Science and Technology, Tsukuba, Ibaraki 305-8563, Japan;; ^e^Graduate School of Science, Hiroshima University, Higashi-Hiroshima, Hiroshima 739-8526, Japan;; ^f^Graduate School of Medical Life Science, Yokohama City University, Yokohama, Kanagawa 230-0045, Japan

**Keywords:** cryo–electron microscopy, nucleosome, chromatin structure

## Abstract

The canonical nucleosome, which represents the major packaging unit of eukaryotic chromatin, has an octameric core composed of two histone H2A-H2B and H3-H4 dimers with ∼147 base pairs (bp) of DNA wrapped around it. Non-nucleosomal particles with alternative histone stoichiometries and DNA wrapping configurations have been found, and they could profoundly influence genome architecture and function. Using cryo–electron microscopy, we solved the structure of the H3-H4 octasome, a nucleosome-like particle with a di-tetrameric core consisting exclusively of the H3 and H4 histones. The core is wrapped by ∼120 bp of DNA in 1.5 negative superhelical turns, forming two stacked disks that are connected by a H4-H4’ four-helix bundle. Three conformations corresponding to alternative interdisk angles were observed, indicating the flexibility of the H3-H4 octasome structure. In vivo crosslinking experiments detected histone–histone interactions consistent with the H3-H4 octasome model, suggesting that H3-H4 octasomes or related structural features exist in cells.

## Significance

Genetic information is stored in chromatin, with nucleosomes as the basic unit. A typical nucleosome comprises an octameric core, consisting of two copies of the histone H2A-H2B dimers and H3-H4 dimers, wrapped by one and a half turns of DNA. In the present study, we determined the structure of an unconventional nucleoprotein particle called the H3-H4 octasome, which has a core composed of four dimers of human H3-H4 without H2A-H2B, with DNA wrapped around this core in a nucleosome-like configuration. Histone–histone interactions observed in the human H3-H4 octasome structure were found in yeast. The incorporation of H3-H4 octasomes into the eukaryotic genome will likely alter chromatin structure and dynamics, representing a paradigm shift in our understanding of epigenome regulation.

The genomic DNA of eukaryotes is packaged into chromatin, in which the basic unit is the nucleosome core particle, consisting of an octameric core composed of two copies each of H2A-H2B and H3-H4 dimers wrapped by a 145- to 147-base pair (bp) DNA fragment with 1.7 left-handed superhelical turns (1). The nucleosome limits the accessibility of the underlying DNA sequence and generally inhibits the binding of sequence-specific factors. Nucleosomes are structurally heterogeneous. The core histones can be replaced by histone variants or altered with covalent modifications, generating a repertoire of structurally distinct nucleosomes along the chromatin with diverse biophysical and biochemical properties that contribute to the regulation of chromosome structure and nuclear activities (2–4).

While the nucleosome represents the major histone-DNA assembly in cellular chromatin, histone-DNA complexes with alternative stoichiometries, such as subnucleosomes, have also been observed (5, 6). For example, the hexasome, which has one less H2A-H2B dimer than the canonical nucleosome, can form when the transcription machinery traverses a nucleosome during elongation (7, 8). Indeed, hexasomes are detected throughout the genome (9, 10). The tetrasome, which contains an (H3-H4)_2_ tetramer core without H2A-H2B, is an intermediate structure involved in nucleosome formation (11). During this step, two H3-H4 dimers associate with a DNA fragment to form a tetrasome, followed by the deposition of two H2A-H2B dimers in a process mediated by histone chaperones in vivo (12, 13).

The H3-H4 tetrasome, which is associated with ∼70 bp of DNA, can be reconstituted by combining (H3-H4)_2_ tetramers and DNA at an equimolar ratio (2, 14). At higher protein-to-DNA ratios, H3-H4 can form a nucleosome-size particle consisting of an octameric H3-H4 core wrapped by ∼130 bp of DNA (15, 16). This nucleosome-like particle, called the H3-H4 octasome hereafter, exhibits a bead-like structure with a diameter comparable to that of the nucleosome, as evidenced by early electron microscopy (EM) studies and a recent atomic force microscopy analysis (2, 17, 18). The (H3-H4)_2_ tetramer alone is sufficient to be positioned around the center of the *Lytechinus variegatus* 5S rRNA gene (rDNA) sequence, a naturally occurring nucleosome positioning sequence (14). Interestingly, when reconstituted with a 2:1 tetramer-to-DNA concentration, the tetramers redistribute equally to the two halves of the 5S rDNA sequence to form a “di-tetrasome” particle, consistent with the H3-H4 octasome configuration (19).

In this study, we determined cryo-EM structures of the H3-H4 octasome. The data revealed that the core of the H3-H4 octasome is composed of two (H3-H4)_2_ tetramers, wrapped by ∼120 bp of DNA in 1.5 left-handed superhelical turns. Along the dyad axis where the two tetramers meet, two H4 molecules form a four-helix bundle (FHB). To assess the biological relevance of the H3-H4 octasome structure, we interrogated yeast chromatin with in vivo crosslinking assays and detected histone–histone interactions observed in the human H3-H4 octasome structure. The implications of the H3-H4 octasome on chromatin architecture and genome function are discussed.

## Results

### Reconstitution of the H3-H4 Octasome.

**Fig. 1.**
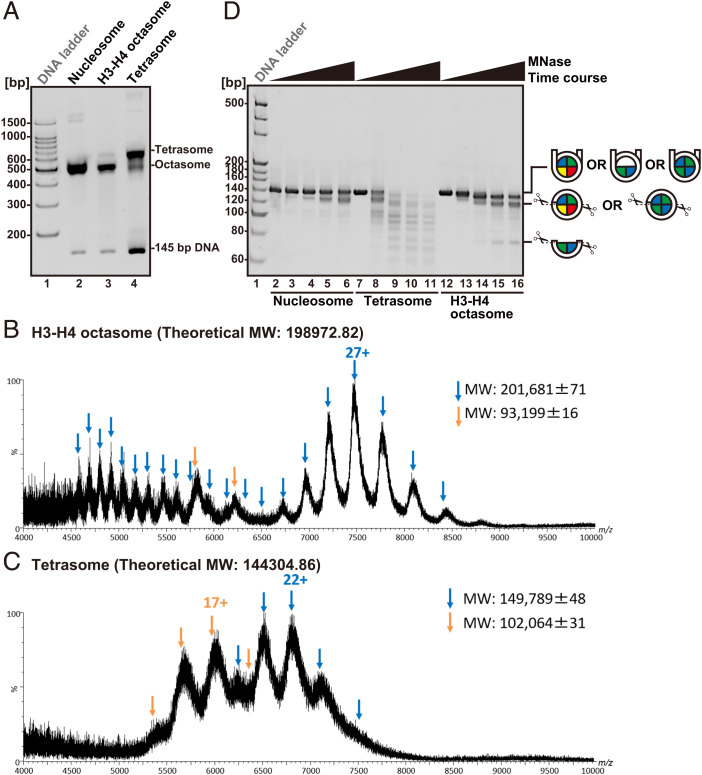
Reconstitution of the H3-H4 octasome. (*A*) Purified nucleosomes, H3-H4 octasomes, and tetrasomes reconstituted with human histones and a 145-bp Widom 601 positioning sequence were analyzed by native PAGE with ethidium bromide (EtBr) staining. (*B* and *C*) ESI mass spectra of H3-H4 octasomes (*B*) and tetrasomes (*C*). Blue and orange arrows indicate multiply charged ions of the nucleoproteins and double-stranded DNA, respectively. Numeric values indicate the charge states of the dominant peaks for individual species. m/z, mass-to-charge ratio; MW, molecular weight. (*D*) The indicated nucleoproteins were treated with MNase for 0, 3, 6, 18, and 36 min from left to right. The DNA products were dissociated from the histones and analyzed by native PAGE with EtBr staining.

Nucleoprotein complexes with human histones H3 and H4 and a 145-bp DNA fragment containing the Widom 601 positioning sequence were reconstituted by the salt dialysis method (20). Two distinct protein complexes were detected by native polyacrylamide gel electrophoresis (PAGE) (Fig. 1*A*). Electrospray ionization mass spectrometry (ESI-MS) revealed that the molecular weights of the nucleoproteins corresponding to the upper and lower bands were 149,789 and 201,681, respectively. These values are consistent with but greater than the theoretical molecular weights of the H3-H4 tetrasome (144,305) and the H3-H4 octasome (198,973) formed on the 145-bp Widom DNA (Fig. 1 *B* and *C* and *SI Appendix*, Fig. S1). The discrepancies are likely due to the replacement of protons in the phosphate groups of the DNA by monovalent cations, such as K^+^, Na^+^, and NH_4_^+^, which were present in the sample buffer and concentrated during the ionization step (21).

To assess the extent of histone-DNA contacts within the H3-H4 octasome, nuclease sensitivity assays were performed with H3-H4 octasomes, along with canonical nucleosomes and H3-H4 tetrasomes as controls (Fig. 1*D*). Micrococcal nuclease (MNase) is an endo/exo nuclease that preferentially digests DNA detached from the histone surface but not DNA stably wrapped around histones, as in the nucleosome (22). Time courses of MNase digestion revealed that canonical nucleosomes protect a dominant DNA species at ∼145 bp, corresponding to the stably wrapped nucleosomal DNA, and an ∼120-bp species at later time points, consistent with partial unwrapping of the DNA ends (23) (Fig. 1 *D*, lanes 2 to 6). By contrast, the H3-H4 tetrasome was highly susceptible to MNase attack, indicating substantial exposure of its DNA to the solvent (Fig. 1 *D*, lanes 7 to 11). The DNA was more strongly protected in the H3-H4 octasome compared to the H3-H4 tetrasome, although the full-length 145-bp DNA fragment was progressively trimmed to ∼130 bp and further to ∼120 bp and ∼70 bp at later time points (Fig. 1 *D*, lanes 12 to 16). The DNA protection pattern of the H3-H4 octasome suggested that the octasomal histone core is stably wrapped by a DNA fragment, but in a manner that differs from that of the canonical nucleosome.

### Overall Structure of the H3-H4 Octasome.

**Fig. 2.**
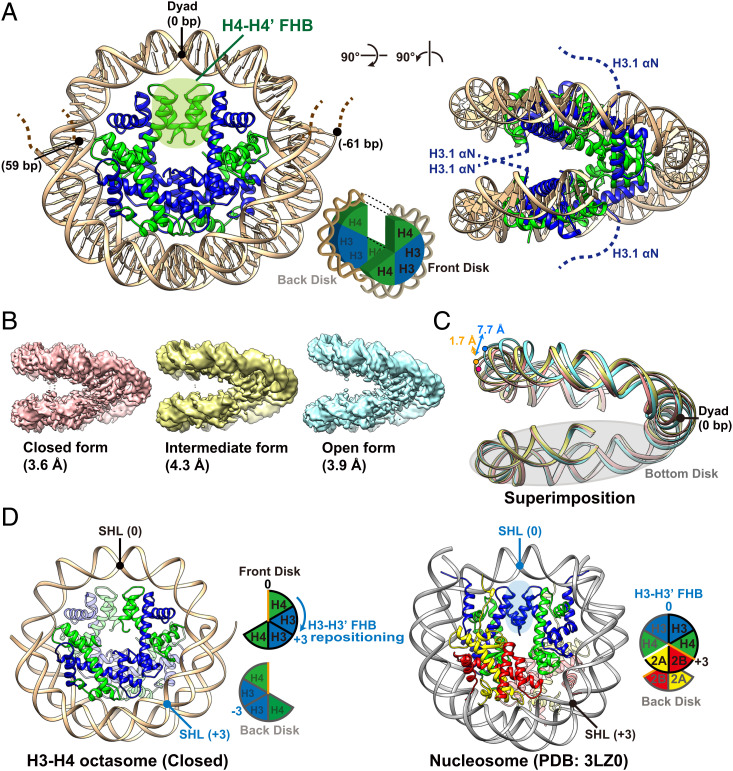
Cryo-EM structures of the H3-H4 octasome. (*A*) The cryo-EM structure of the H3-H4 octasome (closed form). The electron densities of the *N*-terminal regions (amino acid residues 1 to 58) of H3.1 and the DNA termini were not observed (indicated by dashed lines). (*B*) Cryo-EM densities of the H3-H4 octasome in the closed (*Left*), intermediate (*Middle*), and open (*Right*) forms. (*C*) Alternative conformations of the H3-H4 octasome superimposed by aligning the bottom disk. The dots indicate the positions of G39 in the DNA strand of the H3-H4 octasome (pink: closed, orange: intermediate, blue: open). The double arrow indicates the difference of the measured distances between the DNA gyres in these conformations. (*D*) Ribbon models of the H3-H4 octasome (*Left*) and the canonical nucleosome (*Right*). In the H3-H4 octasome, the two H3-H3’ FHBs are positioned three helical turns away from the H3-H4 octasomal dyad at the SHLs +3 and −3. Orange lines in the schematic representations of the histone core assemblies indicate the H4-H4’ interface and the H4-H2B interface of the H3-H4 octasome and the nucleosome, respectively. The white line between H4 and H2A in the nucleosome schematic emphasizes that the two proteins are not connected.

The structure of the H3-H4 octasome was determined by cryo-EM (Fig. 2*A*). The purified H3-H4 octasome sample was analyzed with a 300-kV electron microscope. Approximately 1.43 million particles related to the H3-H4 octasome were identified from 5,517 electron micrographs (*SI Appendix*, Figs. S2 and S3). Single-particle analysis identified three well-resolved structures, indicating that the H3-H4 octasome exists in alternative conformations. The three structures, namely the open, closed, and intermediate forms, were similar but differed by the clamshell opening angle of the two stacked disks formed by the symmetrical halves of the H3-H4 octasome (Fig. 2*B* and *SI Appendix*, Figs. S2 and S3). The closed form, with a resolution of 3.6 Å, was best resolved (Fig. 2*A*). The structure showed that the H3-H4 octasome has a core composed of two (H3-H4)_2_ tetramers, forming a left-handed ramp allowing ∼120 bp of DNA to wrap around the core 1.5 times (Fig. 2*A*). Each (H3-H4)_2_ tetramer engaged an ∼60-bp DNA segment on either side of the dyad to form a disk. A novel H4-H4’FHB, assembled by the helix–loop–helix regions of the two inward-facing H4 histones, connected the two disks (Fig. 2*A*). In the nucleosome, the H3-H3’ FHB of the (H3-H4)_2_ tetramer coincided with the nucleosomal dyad; however, in the H3-H4 octasome, there were two H3-H3’ FHBs, and they were positioned at the superhelical locations (SHLs) +3 and −3 (Fig. 2*D*) on either side of the dyad (SHL 0).

**Fig. 3.**
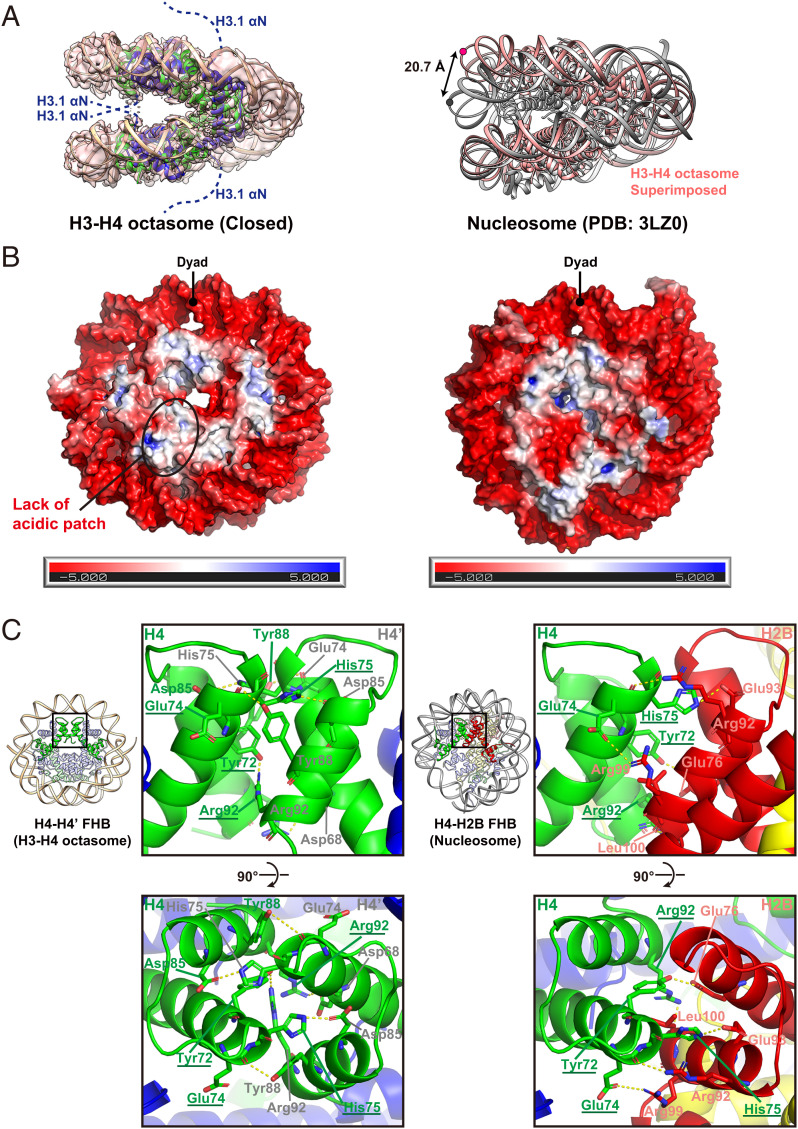
Structural comparison of the H3-H4 octasome and nucleosome. (*A*) The cryo-EM density map superimposed on the ribbon model of the closed form of the H3-H4 octasome (*Left*). The ribbon model of the H3-H4 octasome superimposed on that of the nucleosome (*Right*). The pink dot indicates the positions of G39 in the DNA strand of the H3-H4 octasome, while the black dot indicates T39 in the DNA strand of the nucleosome. The double arrow indicates the difference of the measured distances between the DNA gyres in those complexes. (*B*) The electrostatic potentials of the atomic surfaces of the H3-H4 octasome (*Left*) and the nucleosome (*Right*). (*C*) Close-up views of the H4-H4’ FHB interface of the H3-H4 octasome (*Left*) and the H4-H2B FHB interface of the nucleosome (*Right*). Common residues utilized in both interactions are underlined.

The open and intermediate forms of the H3-H4 octasome structure were determined at 3.9 Å and 4.3 Å resolution, respectively (*SI Appendix*, Figs. S2 and S3). The histone core arrangement and DNA wrapping configuration were similar to those of the closed form, but differed in the distances of the opening measured between the two DNA gyres at the farthest points from the dyad, which were 7.7 Å and 1.7 Å wider in the open and intermediate forms, respectively (Fig. 2*C*). By contrast, the opening between the two disks of the nucleosome was much smaller. When the lower disk of the nucleosome was superimposed on the H3-H4 octasome, the upper disk of the H3-H4 octasome showed an ∼20 Å outward displacement relative to the nucleosome (Fig. 3 *A*, *Right*).

Further comparison of the H3-H4 octasome with the canonical nucleosome revealed additional H3-H4 octasome-specific features. First, the H3-H4 octasome lacked the acidic patch provided by H2A and H2B, which functions as a docking site for a variety of nucleosome binding proteins (24) (Fig. 3*B*). Second, the αN regions of the two inward-facing H3 histones were predicted to occupy the interdisk space of the H3-H4 octasome; however, the expected alpha helical structures were not visible, suggesting that the H3 αN regions in the interdisk space are dynamic (Fig. 3*A*). The remaining two H3 αN regions exposed on the outer surfaces of the H3-H4 octasome were also unstructured. Finally, the FHB at the octasomal H4-H4’ interface resembled the one at the nucleosomal H4-H2B interface (*SI Appendix*, Fig. S4 *A–C*). For example, in the nucleosome, the sidechains of H4 Tyr72, Glu74, His75, and Arg92 interacted with those of H2B Glu76, Arg99, Glu93, and Leu100, respectively. In the H3-H4 octasome, the same H4 sidechains were oriented in a similar manner, but they interacted with the sidechains of Arg92, Tyr88, Asp85, and Asp68 on the opposite H4’ instead (Fig. 3*C*).

### Detection of H3-H4 Octasome-Specific Interactions In Vivo.

**Fig. 4.**
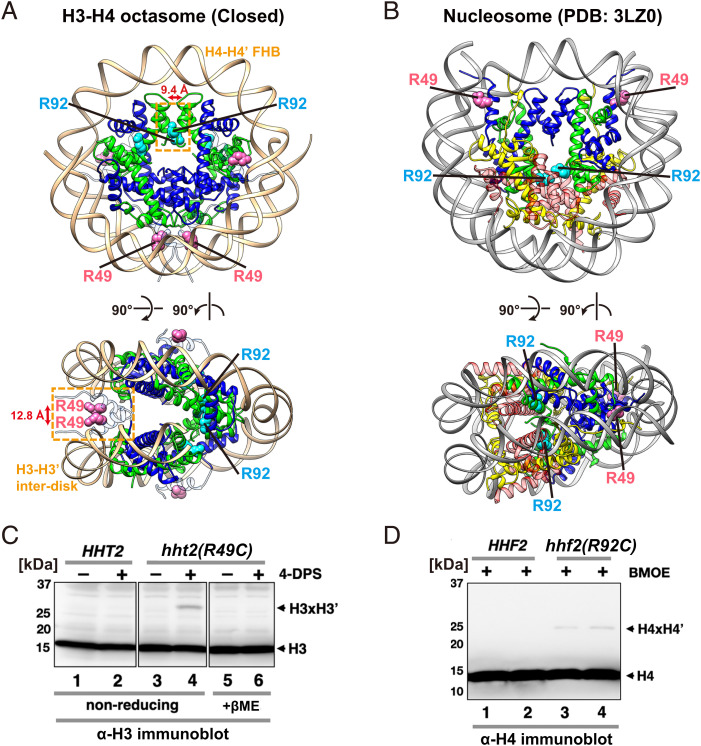
Site-specific crosslinking of the H3-H4 octasome in vivo. (*A*) Locations of the H3 Arg49 and H4 Arg92 residues in the H3-H4 octasome, shown as pink and blue spheres, respectively. The unstructured H3.1 αN helices of the H3-H4 octasome (white) were modeled by superimposition with the H3.1 structure of the nucleosome (PDB ID: 3LZ0). The red double arrow indicates the approximated Cβ-Cβ’ distance between R49 cysteines used for crosslinking. (*B*) Locations of H3 Arg49 and H4 Arg92 in the nucleosome. (*C*) In vivo disulfide crosslinking analysis of the H3-H3′ interdisk interface. Protein extracts from *HHT2* and *hht2(R49C)* yeast cells, with or without 4-DPS treatment, were fractionated by SDS-PAGE and analyzed by immunoblotting with an anti-H3 antibody. Lanes 1 to 4, no βME (nonreducing) and lanes 5 and 6, + βME (reducing). (*D*) In vivo crosslinking of the H4-H4’ FHB interface in the *hht2(R92C)* mutant using BMOE. Protein extracts were analyzed by nonreducing anti-H4 immunoblotting as in *C*. Lanes 3 and 4 represent two individual transformants of *hhf2(R92C)*.

To assess the biological relevance of the H3-H4 octasome, in vivo crosslinking experiments were performed in *Saccharomyces cerevisiae* to determine whether the histone-histone contacts predicted by the H3-H4 octasome structure occur inside cells. Arg49 of yeast H3 was substituted with cysteine so that the inward-facing H3 R49C sites would be close enough to allow disulfide crosslinking at the interdisk interface of the H3-H4 octasome (Fig. 4 *A*, *Bottom*; Cβ-Cβ’ of 12.8 Å). However, these same sites should be too far apart to crosslink in the nucleosome (Fig. 4*B*; Cβ-Cβ’ of 54.3 Å) (25).

The yeast histone H3 gene *HHT2* with or without R49C was expressed as the sole source of H3, under endogenous promoter control on a low-copy plasmid (26). *hht2(R49C)* cells were viable, albeit slow growing, indicating that the *hht2(R49C)* allele is at least partially functional (*SI Appendix*, Fig. S5 *A* and *B*). After *HHT2* and *hht2(R49C)* cells were treated with 4,4'-dipyridyl disulfide (4-DPS), a cell-permeable oxidant, total proteins were analyzed by anti-H3 immunoblotting (25) (Fig. 4*C*). An ∼30-kDa band was observed in the *hht2(R49C)* strain, but not *HHT2* (Fig. 4 *C*, lanes 2, 4). This 30-kDa band was absent when the extracts were pretreated with beta-mercaptoethanol (βME), confirming that the H3 adduct had a cystine linkage (Fig. 4 *C*, lane 6). In vivo crosslinking was also observed for *hht2(V46C)* and *hht2(A47C)* (within the unstructured H3 αN region at the interdisk interface) but not *hht2(S102C)* (within the α2 helix of H3), consistent with the H3-H4 octasome structure (*SI Appendix*, Fig. S5 *C–E*).

To verify that the 30-kDa band was due to a crosslinked adduct between two H3 molecules (15 kDa each) but not H3 with another cysteine-containing protein, we cotransformed a V5-tagged *hht1(R49C)* with an untagged *hht2(R49C)* into yeast to generate a “hybrid” strain heterozygous for the tag. Anti-H3 and anti-V5 immunoblotting analyses collectively showed that the hybrid strain has three H3 crosslinking bands, confirming that the crosslinked species in *hht2(R49C)* are between two H3 molecules (*SI Appendix*, Fig. S5*F*).

As a second test for the in vivo presence of the H3-H4 octasome, the unique H4-H4’ FHB interaction was targeted using a yeast strain in which the histone H4 gene *HHF2* contains a cysteine substitution at Arg92 (*SI Appendix*, Fig. S5*G*). In the H3-H4 octasome, the two H4 R92C sites on the FHB were 9.4 Å apart and facing each other (Fig. 4 *A*, *Top*). By contrast, in the nucleosome, the same sites were 16.8 Å apart (Cβ-Cβ’) and facing away from each other (Fig. 4*B*). The bifunctional sulfhydryl crosslinker bis-maleimidoethane (BMOE), instead of 4-DPS, was used to crosslink the R92C sites because the FHB structure lacks the structural flexibility required for disulfide crosslinking (27, 28). To validate the specificity of BMOE, we first treated *hht2(V46C)* cells with BMOE or 4-DPS. An ∼30-kDa H3-H3’ adduct was observed in both cases, suggesting that BMOE successfully detected the interdisk H3-H3’ interaction (*SI Appendix*, Fig. S5*H*). However, unlike the 4-DPS–induced cystine, the linkage induced by BMOE was not cleaved by βME. Importantly, when *hhf2(R92C)* cells were treated with BMOE, an adduct consistent with H4-H4’ crosslinking was observed (Fig. 4 *D*, lanes 3 and 4), but no crosslinking was detected in wild-type cells (Fig. 4 *D*, lanes 1 and 2). This result suggests that the H4-H4’ FHB interaction occurred in vivo.

**Fig. 5.**
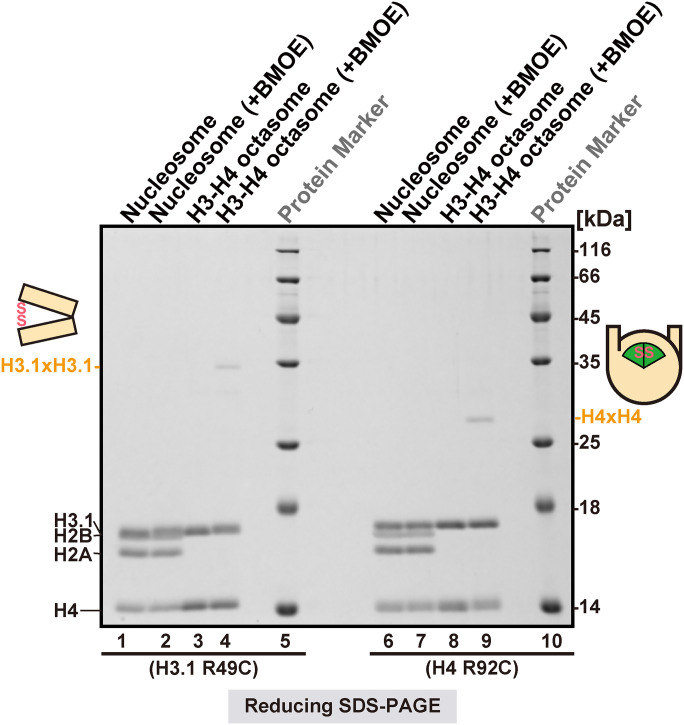
Site-specific crosslinking of the H3-H4 octasome in vitro. BMOE crosslinking analysis of reconstituted nucleosomes and H3-H4 octasomes containing human histones H3.1 R49C (lanes 1 to 4) or H4 R92C (lanes 6 to 9) as the sole cysteines. The schematic on the left is a side view of the H3-H4 octasome, which indicates the position of the crosslinked H3.1-H3.1’, while the schematic on the right is a top view of the H3-H4 octasome, which indicates crosslinked H4-H4’. The nucleoproteins were treated with BMOE and subjected to reducing SDS-PAGE and CBB staining.

To confirm that the crosslinking adducts observed in *hht2(R49C)* and *hhf2(R92C)* cells were due to interactions specific to H3-H4 octasomes but not nucleosomes, we reconstituted nucleoprotein assemblies containing human histone H3.1 bearing R49C or H4 bearing R92C and subjected them to in vitro crosslinking analyses using BMOE (Fig. 5). The naturally occurring Cys96 and Cys110 in H3.1 were replaced with serine and alanine, respectively, to avoid spurious crosslinking (*SI Appendix*, Fig. S6*A*). When H3-H4 octasomes containing the H3 R49C substitution were incubated with BMOE, an ∼30-kDa adduct was observed with a reciprocal decrease of the monomeric H3 species, suggesting that the opposing H3 R49C sites were within crosslinking distance (Fig. 5 and *SI Appendix*, Fig. S6*C*). However, no observable crosslinking adducts were detected for H3 R49C nucleosomes. Similarly, when H3-H4 octasomes bearing the H4 R92C site were treated with BMOE, an ∼28-kDa crosslinking adduct was observed with a reciprocal decrease of monomeric H4 (Fig. 5 and *SI Appendix*, Fig. S6*C*). Again, no crosslinking adducts were observed for H4 R92C nucleosomes.

Another explanation for the observed H3-H3’ or H4-H4’ crosslinking is that when consecutive cysteine-modified nucleosomes are organized in arrays, cysteine probes on neighboring nucleosomes may interact to allow crosslinking to occur. However, this is not the case, at least in vitro. We assembled trinucleosomes (separated by two 22-bp linkers) containing the H3 R49C or the H4 R92C substitution and subjected these short nucleosomal arrays to BMOE-induced crosslinking (*SI Appendix*, Fig. S6 *G* and *H*). No detectable crosslinking adducts were found under conditions that permitted crosslinking of H3-H4 octasomes with the same cysteine substitutions. Finally, 4-DPS was also used to induce disulfide crosslinking of H3-H4 octasomes bearing the H3 R49C site (*SI Appendix*, Fig. S6 *D–F*). An ∼30-kDa disulfide adduct was detected for the H3 R49C-H4 octasomes but not the H3 R49C nucleosomes. Note that crosslinked adducts were formed even in the absence of 4-DPS, presumably by atmospheric oxidation.

## Discussion

Nucleosomes act as a physical barrier for DNA-binding proteins, which regulate genomic DNA functions such as transcription, replication, recombination, and repair (29). In these processes, nucleosomes must be disassembled and reassembled. Misregulation of these cycles is linked to various diseases, including cancer (30). Subnucleosomes and nucleosome-like particles are dynamic structures that emerge and/or function during these cycles (31, 32). In the present study, we determined the cryo-EM structures of a nucleosome-like particle, the H3-H4 octasome, which has unique features not found in the canonical nucleosome. In these structures, an ∼120-bp DNA segment and four copies of H3-H4 dimers formed a stable core particle without H2A and H2B.

The H3-H4 octasome surface lacks the common foothold known as the acidic patch, which functions as an anchoring site for nucleosome-binding proteins, including histone modifiers and nucleosome remodelers (24). Thus, the presence of H3-H4 octasomes could interfere with the propagation of epigenetic marks and the spacing of nucleosomal arrays. Notably, the acidic patch serves as the docking site for the H4 N-terminal tail of a neighboring nucleosome, an interaction critical for chromatin compaction (1, 33). The absence of the acidic patch in the H3-H4 octasome could therefore interrupt chromatin fiber formation. In addition, the unstructured *N* termini on the two outer H3 molecules are expected to occupy the spaces above and below the H3-H4 octasome disks, interfering with stacking interactions between nucleosomes. Thus, chromatin fibers punctuated with H3-H4 octasomes could contribute to alternative higher-order conformations that may influence a wide variety of genomic functions.

The H3-H4 octasome exhibits larger interdisk spacing between DNA superhelical gyres compared to the nucleosome. This is probably due to the lack of the H2A-mediated L1-L1’ interaction and the steric repulsion of the extended unstructured *N* termini on the two inward-facing H3 molecules. The interdisk spacing appears to be dynamic, as evidenced by the alternative conformations of the H3-H4 octasome structure. The wider interdisk space and the dynamic nature of the clamshell structure suggest that the H3-H4 octasome could provide greater access to DNA-binding factors, such as pioneer factors, which prefer to interact with the DNA along the gyre (34).

We note that the observed in vivo crosslinking of cysteine-modified histones is not proof for the existence of discrete H3-H4 octasome particles within the cell. An alternative explanation is that the observed crosslinking may represent interactions of subnucleosomal particles, such as hexasomes, which are distributed broadly across the genome and are robust substrates for chromatin remodelers (9, 10). It is conceivable that when two hexasomes slide into each other, the interactions of the two sides with an exposed H4 could generate an interface that is structurally similar to the interdisk interface of the H3-H4 octasome. Although this configuration would have two H2A-H2B dimers on the terminal sides of the H3-H4 octasome, our data do not rule out such a possibility.

In the H3-H4 octasome, the formation of the H4-H4’ FHB involves the conserved H4 residues Asp68, Tyr72, Glu74, His75, Asp85, Tyr88, and Arg92 in the α2-L2-α3 region (*SI Appendix*, Fig. S6*B*). The fact that H4 can pair with another H4 (as opposed to H2B in the nucleosome) in vitro was previously noted by the Richmond laboratory, but not structurally resolved until now (19).

The two outward-facing H4 molecules in the H3-H4 octasome each have an unpaired α2-L2-α3, raising the possibility that additional (H3-H4)_2_ tetramers can stack onto the H3-H4 octasome and polymerize along the DNA. In fact, a previous report suggested the stacking of up to four (H3-H4)_2_ tetramers based on a minor but distinct peak observed in an MNase sensitivity assay of nucleohistone assemblies reconstituted with H3 and H4 (15).

The eukaryotic (H3-H4)-exclusive fiber is perhaps not unlike the archaeal chromatin fiber, in which DNA wraps around a polymer of archaeal histone homodimers to form a quasi-continuous superhelical structure (35–38). However, the eukaryotic (H3-H4)-exclusive fiber would likely exhibit a strong bend. Comparisons of the H3-H4 octasome with the archaeal nucleosome-like particle, known as the archaeasome, indicated a much wider DNA gyre separation (12.7 Å). This is because the interdisk space of the H3-H4 octasome is occupied by the two extended, unstructured H3 *N* termini, whereas in the archaeasome, the disks are held together by stacking interactions, contributed in part by the interdisk L1-L1’ contact between the dimers at positions N and N+3 (36). Interestingly, a recent study that combined cryo-EM and molecular simulation analyses indicated that archaeal chromatin is not a straight rod, but perhaps more slinky-like (35). In fact, the archaeasome disks can open, like a clam, with a 90° angle (36). Therefore, eukaryotic chromatin may share more similarity with archaeal chromatin than previously thought.

In summary, the structural insights of the H3-H4 octasome provided in this study underscore how eukaryotes may utilize alternative histone arrangements to modulate chromatin structure and dynamics. The next major challenge is to understand how H3-H4 octasomes interact with nuclear factors to modulate genomic functions.

## Materials and Methods

### Purification of DNA Fragments.

The 145-bp DNA fragment derived from the Widom 601 sequence (ATCAGAATCCCGGTGCCGAGGCCGCTCAATTGGTCGTAGACAGCTCTAGCACCGCTTAAACGCACGTACGCGCTGTCCCCCGCGTTTTAACCGCCAAGGGGATTACTCCCTAGTCTCCAGGCACGTGTCAGATATATACATCGAT) was prepared as previously described (39). Tandem repeats of the DNA fragment were inserted into the pGEM-T Easy vector (Promega). The plasmid was amplified in *Escherichia coli* cells and purified. The DNA fragment was cleaved by digestion with *Eco*RV (Takara), and the vector DNA region was removed by precipitation with polyethylene glycol 6,000. The DNA fragment was further purified by TSKgel DEAE-5 PM (TOSOH) column chromatography. The DNA fragment for trinucleosome reconstitution contains three Widom 601 sequences separated by two 22-bp linker regions and was prepared as previously described (40).

### Preparation of Human Histones, Histone Complexes, and Nucleosomes.

The human histones H2A, H2B, H3.1, H3.1(C96S, C110A), H3.1(C96S, C110A, R49C), H4, and H4(R92C) were expressed and purified as previously described (7). The histone octamer and the H3-H4 tetramer were refolded by dialysis and purified by gel filtration chromatography on a HiLoad 16/600 Superdex 200 pg column (Cytiva). H3-H4 octasomes and tetrasomes were reconstituted by mixing H3-H4 tetramers with the Widom 601 DNA (145 bp) (20), followed by refolding using the salt dialysis method (7). More specifically, the purified DNA was mixed with H3-H4 tetramers at a DNA-to-histone molar ratio of 1:2.2 (for H3-H4 octasomes) or 1:1 (for tetrasomes) in 2 M KCl buffer. The nucleoprotein mixtures were then dialyzed against buffer containing 10 mM Tris⋅HCl (pH 7.5), 2 M KCl, 1 mM ethylenediaminetetraacetic acid (EDTA), and 1 mM dithiothreitol (DTT). The KCl concentration was gradually reduced to 0.25 M by exchanging the buffer with a peristaltic pump. The resulting H3-H4 octasomes (but not tetrasomes) were incubated at 55 °C for 2 h and then purified by 6% nondenaturing PAGE using a Prep Cell apparatus (Bio-Rad). The 485-bp trinucleosome with the 22-bp linker was prepared as previously described (40).

### MNase Treatment Assay.

Nucleosomes with the 145-bp DNA (200 ng each) were mixed with 0.01 unit/μL MNase (Takara) and incubated at 37 °C for 0, 3, 6, 18, and 36 min in buffer containing 50 mM Tris⋅HCl (pH 7.5), 2.5 mM CaCl_2_, 1.9 mM DTT, and 50 mM NaCl. MNase reactions were quenched by buffer containing 20 mM Tris⋅HCl (pH 7.5), 20 mM EDTA, 0.5 mg/mL proteinase K, and 0.25% sodium dodecyl sulfate (SDS). The resulting DNA fragments were subjected to phenol/chloroform extraction and ethanol precipitation and then analyzed by 10% native PAGE in 0.5x TBE buffer (45 mM Tris base, 45 mM boric acid, and 1 mM EDTA).

### Native ESI-MS.

ESI mass spectra were acquired with a Triwave SYNAPT G2 high-definition mass spectrometer (Waters) with a nanoESI source (41–44). For native ESI-MS, the H3-H4 octasome and tetrasome were dialyzed against 50 mM ammonium acetate (NH_4_OAc), and their concentrations were adjusted to 2 µM. A low flow rate nanoESI procedure was used to ionize the samples (45). Nanospray tips were prepared in-house by pulling borosilicate glass capillaries (using a P-97 capillary puller; Sutter Instruments) and processed to ∼5 µm inner diameter (using a microforge MF-900 instrument; Narishige). The tips were coated with gold using a sputter coater. To observe the ions of the samples, the back pressure was adjusted to ∼5 mbar with a SpeediValve. The following parameters were used for analysis: 0.70- to 1.0-kV capillary voltage, 20-V sampling cone voltage, 4-V trap collision energy, and 3.0 mL/min trap argon gas flow rate. The mass spectra were acquired for *m/z* 2,000 to 14,000 and calibrated with (CsI)nCs^+^ ions. The MassLynx version 4.1 software (Waters) was used for data processing and peak integration.

### Cryo-EM Sample Preparation and Data Collection.

To stabilize the purified H3-H4 octasome, the gradient fixation method (GraFix) was performed during sucrose gradient centrifugation (46). A linear gradient was prepared using a Gradient Master instrument (SKB), with low buffer containing 20 mM N-(2-Hydroxyethyl)piperazine-N′-(2-ethanesulfonic acid) titrated with potassium hydroxide to pH 7.5 (Hepes-KOH), 1 mM DTT, and 10% (wt/vol) sucrose and high buffer containing 20 mM Hepes-KOH (pH 7.5), 1 mM DTT, 25% (wt/vol) sucrose, and 3% paraformaldehyde. Centrifugation was performed for 16 h at 27,000 rpm at 4 °C using an SW41 rotor (Beckman Coulter). The fractions containing the H3-H4 octasome were subjected to buffer exchange chromatography using Micro Bio-Spin Columns (Bio-Rad) equilibrated with 20 mM Tris⋅HCl (pH 7.5) with 1 mM DTT.

For cryo-EM specimen preparation, a 2 µL aliquot of the H3-H4 octasome (0.63 mg/mL) was applied to a glow-discharged Quantifoil R1.2/1.3 200-mesh Cu grid and blotted for 8 s under 100% humidity at 12 °C in a Vitrobot Mark IV system (Thermo Fisher Scientific). The grids with the H3-H4 octasome were immediately plunged into liquid ethane. Cryo-EM data collection of the H3-H4 octasome was performed by SerialEM auto acquisition software (47) on a Krios G3i cryo-electron microscope (Thermo Fisher Scientific), operated at 300 kV with a pixel size of 1.05 Å and a defocus range from −1.25 to −2.5 µm. Images of the H3-H4 octasome were recorded at 13.2 e/pix/s with 6-s exposure times on an energy-filtered K3 direct electron detector (Gatan) in the electron counting mode with a slit width of 25 eV, retaining a total of 40 frames with an overall dose of ∼63 electron/Å^2^.

### Cryo-EM Image Processing.

In total, 5,517 movies of the H3-H4 octasome were aligned by MotionCor2 software (48) with dose weighting. The contrast transfer function (CTF) parameters for each micrograph were estimated by CTFFIND4 (49). RELION 3.0 was used to process the images of the H3-H4 octasome sample as follows (50). From 3,922 micrographs, 1,427,558 particles were picked automatically using the two-dimensional (2D) template-based picker function and subjected to reference-free 2D classification to remove bad particles. Subsequently, 990,623 selected particles were used for three-dimensional (3D) classification (with global soft mask applied). The three best classes from the 3D classification were chosen based on the resolution. The structures were subjected to 3D refinement, followed by Bayesian polishing and two rounds of CTF refinement. C2 symmetry was applied to the 3D reconstruction of the H3-H4 octasome. The final resolutions of the refined maps of the closed, open, and intermediate conformations of the H3-H4 octasome were 3.6 Å, 3.9 Å, and 4.3 Å, respectively, and were determined based on the gold standard Fourier Shell Correlation with the 0.143 criterion (51). The final maps of the H3-H4 octasome were normalized with MAPMAN (52) and visualized with University of California, San Francisco (UCSF) Chimera software (53). The processing statistics for the H3-H4 octasome structures are presented in *SI Appendix*, Table S1.

### Model Building.

The structural models of the closed, open, and intermediate forms of the H3-H4 octasome were built from the H3-H4 tetramer and its proximal DNA fragment in the crystal structure of the nucleosome containing *Xenopus laevis* histones and 145-bp Widom 601 DNA (Protein Data Bank [PDB]: 3LZ0), which represented roughly half of the symmetric structure of the H3-H4 octasome (54). The amino acid residues of the histones were adjusted to those of human histones. The model coordinates were refined automatically with phenix.real_space_refine and manually using Coot (55, 56). The DNA sequence of the H3-H4 octasome was estimated based on MNase and restriction enzyme analyses of H3-H4 octasome samples. All structure figures were prepared using UCSF Chimera and PyMOL (Schrödinger; http://www.pymol.org).

### Yeast Plasmids.

Yeast histone expression plasmids were derived from the *HHT1-HHF1 URA3 CEN ARS* plasmid pMS329 (gift from Mitchell Smith, University of Virginia) and the *HHT2-HHF2 TRP1 CEN ARS* plasmid pWZ414-F12 (gift from Rolf Sternglanz, Stony Brook University) (26, 57). The plasmid for *hht1(R49C)* (pEL649) was constructed by digesting pMS329 with *Cla*I and *Age*I to remove a segment of the H3 gene, followed by recombination with a synthetic DNA fragment (Twist Bioscience) containing the R49C mutation, using the Gibson assembly protocol (New England Biolabs). The plasmids for *hht2(R49C)* (pEL629), *hht2(V46C)* (pEL651), *hht2(A47C)* (pEL652), and *hht2(S102C)* were similarly constructed by digesting pWZ414-F12 with *Blp*I and *Bam*HI to remove the H3 fragment followed by recombination with the mutant sequences. The 2xV5-*HHT1* (pEL656) and 2xV5-*hht1(R49C)* (pEL650) plasmids were generated by digesting pMS329 and pEL649, respectively, with *Age*I and *Pme*I followed by recombination with a synthetic fragment containing the N-terminal 2xV5 tag. The *hhf2(R92C)* plasmid (pEL626) was generated by digesting pWZ414-F12 with *Bam*HI and *Nco*I and replacing the H4 fragment with a synthetic DNA fragment containing the R92C mutation. The integrity of all plasmids was confirmed by Sanger sequencing (Genewiz).

### Yeast Strains.

The histone knockout strain YYY67 (gift from R. Sternglanz) was supplemented with pMS329 (26, 58). The *HHT2 TRP1 CEN ARS* plasmid and the mutant variants were introduced into YYY67 by standard yeast transformation. Transformants were selected on synthetic complete media lacking uracil and tryptophan and then seeded onto complete supplement mixture (CSM) media supplemented with 0.1% (wt/vol) 5-fluoroorotic acid (5-FOA) to select against the *URA3* plasmid (26). Survivors of 5-FOA selection represent the wild-type *HHT2* strain (yEL699) and *HHT2* mutant strains used in the immunoblotting analyses in Fig. 4*C* and *SI Appendix*, Fig. S5 *E* and *H*. For the hybrid mutant in *SI Appendix*, Fig. S5*F*, *hht1(R49C)* and *HHT1*, with or without an N-terminal 2xV5 tag, were introduced into yEL699 and yEL705 on *URA3 CEN ARS* plasmids. When the *hht2(R49C)* yEL705 strain was generated, a strong growth defect was observed on the 5-FOA media, but the defect was partially alleviated when the cells were grown anaerobically using the anaerobe pouch system (BD Biosciences, cat# B260683). The growth defect of *hht2(R49C)* was less severe in media without 5-FOA; thus, the cells were grown aerobically for in vivo crosslinking experiments. The *hht2(A47C)* and *hht2(V46C)* cells exhibited milder growth defects compared to the *hht2(R49C)* cells in all growth conditions.

### In Vivo Crosslinking.

In vivo disulfide crosslinking (VivosX) was performed based on a previously reported protocol (25). Briefly, yeast cells were cultured in CSM media at 30 °C to an optical density (OD) (at 600 nm) of ∼0.5 and then treated with 180 µM 4-DPS (in dimethyl sulfoxide [DMSO]) or with an equivalent volume of DMSO for 20 min at 30 °C. The cells (from 5 mL of the culture) were fixed with 20% trichloroacetic acid (TCA), pelleted by centrifugation, and homogenized in 20% TCA by zirconia bead beating using a FastPrep-24 machine. The precipitates were washed with acetone and extracted with 200 µL TUNES-GN buffer (100 mM Tris⋅HCl [pH 7.2], 6 M urea, 10 mM EDTA, 1% SDS, 0.4 M NaCl, 10% glycerol, and 50 mM *N*-ethylmaleimide) at 30 °C for 1 h with vortexing.

For the experiment in Fig. 4*D*, in vivo crosslinking with BMOE was performed using a modified protocol based on a previous report (28). Yeast cells were cultured in yeast extract-peptone-dextrose media at 30 °C to 0.5 OD. A 2.5-OD equivalent of cells was then pelleted by centrifugation. The cells were washed with 625 µL of ice-cold phosphate-buffered saline (PBS) and then incubated with 5 mM BMOE in 62.5 µL PBS for 6 min on ice. To quench the BMOE, the cells were washed twice with 5 mM DTT in 125 µL PBS. TCA fixation and protein extraction were performed as described above. For the experiment in *SI Appendix*, Fig. S5*H*, 5 mM BMOE was added directly to 5 mL of yeast culture (in CSM) at 0.5 OD and incubated for 20 min at 30 °C, and then the cells were fixed with 20% TCA, washed with PBS, and extracted for protein analysis as described for 4-DPS crosslinking.

SDS-PAGE was performed under nonreducing or reducing conditions as previously described (25). H3, H4, and V5 immunoblotting analyses were performed with an anti-H3 antibody (gift from Carl Wu, Johns Hopkins University), an anti-H4 antibody (Active Motif 91296), and an anti-V5 antibody (Fisher Thermo Scientific, 46–0705) at 1:2,000 dilution. The anti-H3 antibody used in *SI Appendix*, Fig. S5*E* was affinity purified. Secondary antibodies conjugated to horseradish peroxidase were used at 1:5,000 dilution. Immunoblotting signals were developed with the ECL Prime reagent (GE Life Sciences, RPN2232) and imaged with an LAS-4010 CCD camera system (GE Life Sciences).

### In Vitro Crosslinking.

Nucleosomes, trinucleosomes, and H3-H4 octasomes were reconstituted with the human H3.1(C96S, C110A) mutant with or without R49C or the wild-type H4 with or without R92C. The resulting nucleoproteins (at 0.8 µM in 20 mM Tris⋅HCl, pH 7.0) were incubated with 80 µM 4-DPS at 25 °C for 30 min, where indicated. For reducing SDS-PAGE, aliquots of the reactions were mixed at a 1:1 ratio with sample buffer containing 100 mM Tris⋅HCl (pH 6.8), 4% SDS, 20% glycerol, 0.2% bromophenol blue, and 200 mM βME. The samples (280 ng each) were heated at 95 °C for 5 min and then analyzed by SDS-PAGE (16% polyacrylamide) with Coomassie Brilliant Blue (CBB) staining. For nonreducing SDS-PAGE, aliquots of the reactions were mixed with a similar sample buffer but without βME and analyzed by SDS-PAGE without prior heating. For BMOE crosslinking, the nucleoproteins were incubated with 80 µM BMOE for 30 min at 25 °C. Trinucleosomes were incubated at 0.27 µM, which is equivalent to the molar concentration of the nucleosome (0.8 µM) used in the in vitro crosslinking reaction for the mononucleosome. Site-specific crosslinking with BMOE was analyzed by SDS-PAGE under reducing conditions. The integrity of the nucleoproteins before and after 4-DPS and BMOE treatment was verified by native PAGE analysis (with 6% polyacrylamide in 0.5x TBE).

## Supplementary Material

Supplementary File

## Data Availability

The cryo-EM reconstructions and atomic models of the H3-H4 octasome have been deposited to the Electron Microscopy Data Bank and the Protein Data Bank under the following accession codes: EMD-33010 and 7X57 for the closed form (59, 60), EMD-33011 and 7X58 for the open form (61, 62), and EMD-33991 and 7YOZ for the intermediate form (63, 64). All data presented in this study are included in the main article or the *SI Appendix*.
