# Supplementary material for: Cryo–electron microscopy structure of the H3-H4 octasome: A nucleosome-like particle without histones H2A and H2B
Source: Proc Natl Acad Sci U S A. 2022 Nov 2;119(45):e2206542119. doi: 10.1073/pnas.2206542119 (PMC9659345; doi:10.1073/pnas.2206542119)
Supplement: Supplementary File [file pnas.2206542119.sapp.pdf]

Supplementary Information for

Cryo-electron microscopy structure of the H3-H4 octasome—a nucleosome-like particle without histones H2A and H2B

Kayo Nozawa, Yoshimasa Takizawa, Leonidas Pierrakeas, Chizuru Sogawa-Fujiwara, Kazumi Saikusa, Satoko Akashi, Ed Luk* and Hitoshi Kurumizaka*
*Corresponding author. Email: kurumizaka@iqb.u-tokyo.ac.jp (HK) or Ed.Luk@stonybrook.edu (EL)

This PDF file includes:

Figures S1-S6

Tables S1-S3

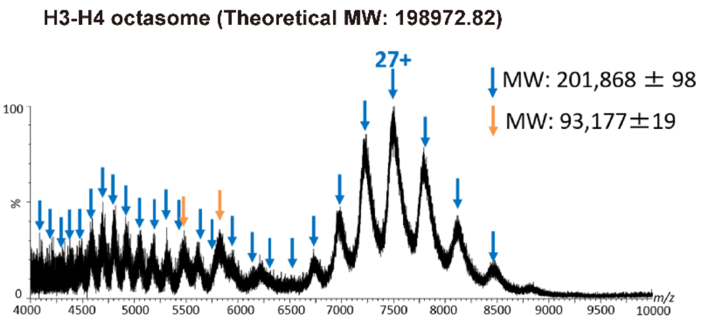

Figure S1. Duplicated measurement of ESI mass spectra of H3-H4 octasome

ESI mass spectra of H3-H4 octasomes. Blue and orange arrows indicate multiply charged ions of the nucleosomes and double stranded DNA, respectively. Numeric values indicate the charge state of the dominant peak for individual species. The increase of mass was 2,895 m/z and similar to that observed in the experiment shown in Figure 1B.

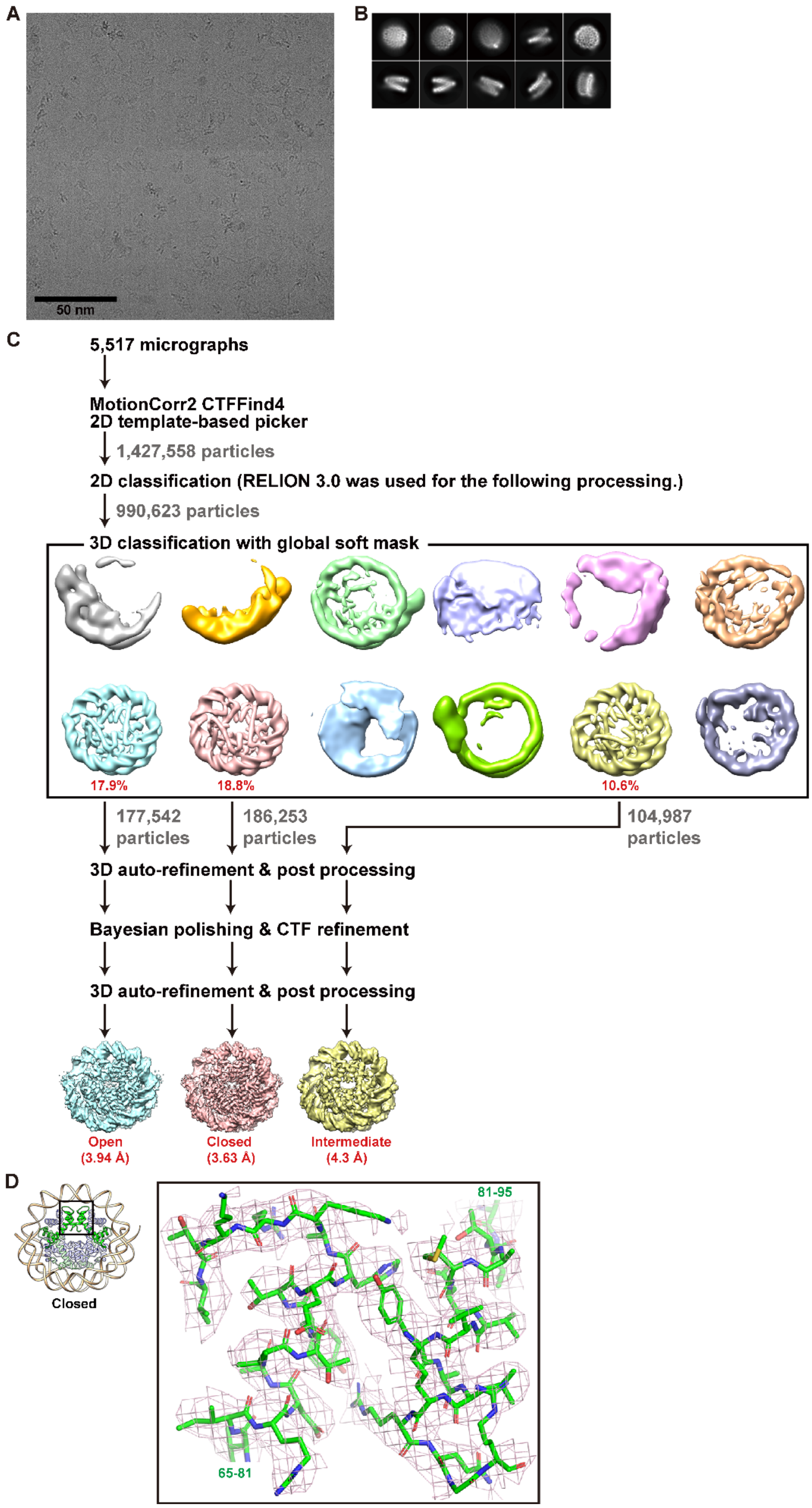
A
50 nm
B
C
5,517 micrographs
MotionCorr2 CTFFind4
2D template-based picker
1,427,558 particles
2D classification (RELION 3.0 was used for the following processing.)
990,623 particles
3D classification with global soft mask
17.9%
18.8%
10.6%
177,542 particles
186,253 particles
104,987 particles
3D auto-refinement & post processing
Bayesian polishing & CTF refinement
3D auto-refinement & post processing
Open (3.94 Å)
Closed (3.63 Å)
Intermediate (4.3 Å)
D
Closed
81-95
65-81

Figure S2. Cryo-EM data collection and image processing of the H3-H4 octasome

(A) Representative micrograph of the cryo-EM dataset. (B) Representative 2D class averages from the reference-free 2D classification calculated after removing bad particles. (C) The workflow of the cryo-EM image processing of the H3-H4 octasome. Box size is 18.9 nm^2. (D) Cryo-EM map of H4-H4' FHD in the H3-H4 octasome in the closed form. The density was calculated from the normalized map and contoured at 5.0σ.

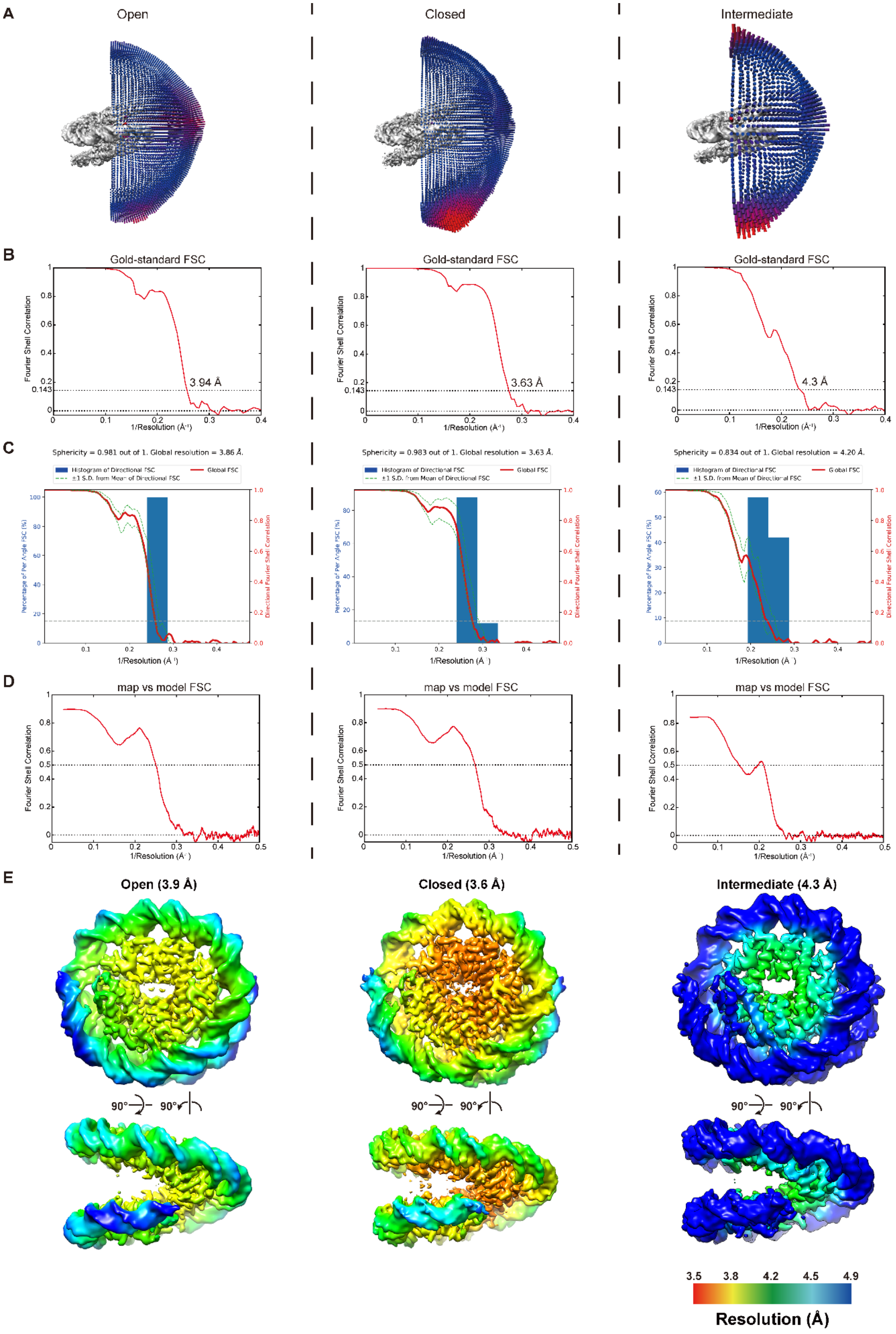
A
Open
Closed
Intermediate
B
Gold-standard FSC
Fourier Shell Correlation
1/Resolution (Å⁻¹)
3.94 Å
3.63 Å
4.3 Å
C
Sphericity = 0.981 out of 1. Global resolution = 3.86 Å.
Sphericity = 0.983 out of 1. Global resolution = 3.63 Å.
Sphericity = 0.834 out of 1. Global resolution = 4.20 Å.
Histogram of Directional FSC
±1 S.D. from Mean of Directional FSC
Global FSC
Percentage of Per Angle FSC (%)
Directional Fourier Shell Correlation
D
map vs model FSC
E
Open (3.9 Å)
Closed (3.6 Å)
Intermediate (4.3 Å)
90°
3.5
3.8
4.2
4.5
4.9
Resolution (Å)

Figure S3. Cryo-EM data validation of the alternative H3-H4 octasome conformations

(A) Euler angular distribution plots of the alternative H3-H4 octasome conformations, calculated using RELION 3.0. Scale shows the number of particles assigned to a particular angle. (B) Gold standard Fourier Shell Correlation (FSC) curves, calculated using RELION 3.0. (C) Directional FSC plots for the reconstructions calculated on the 3DFSC server(1). (D) Map-to-model FSC curves calculated with Phenix. (D) Local resolution maps for the open, closed, and intermediate forms of the H3-H4 octasome, calculated with RELION 3.0.

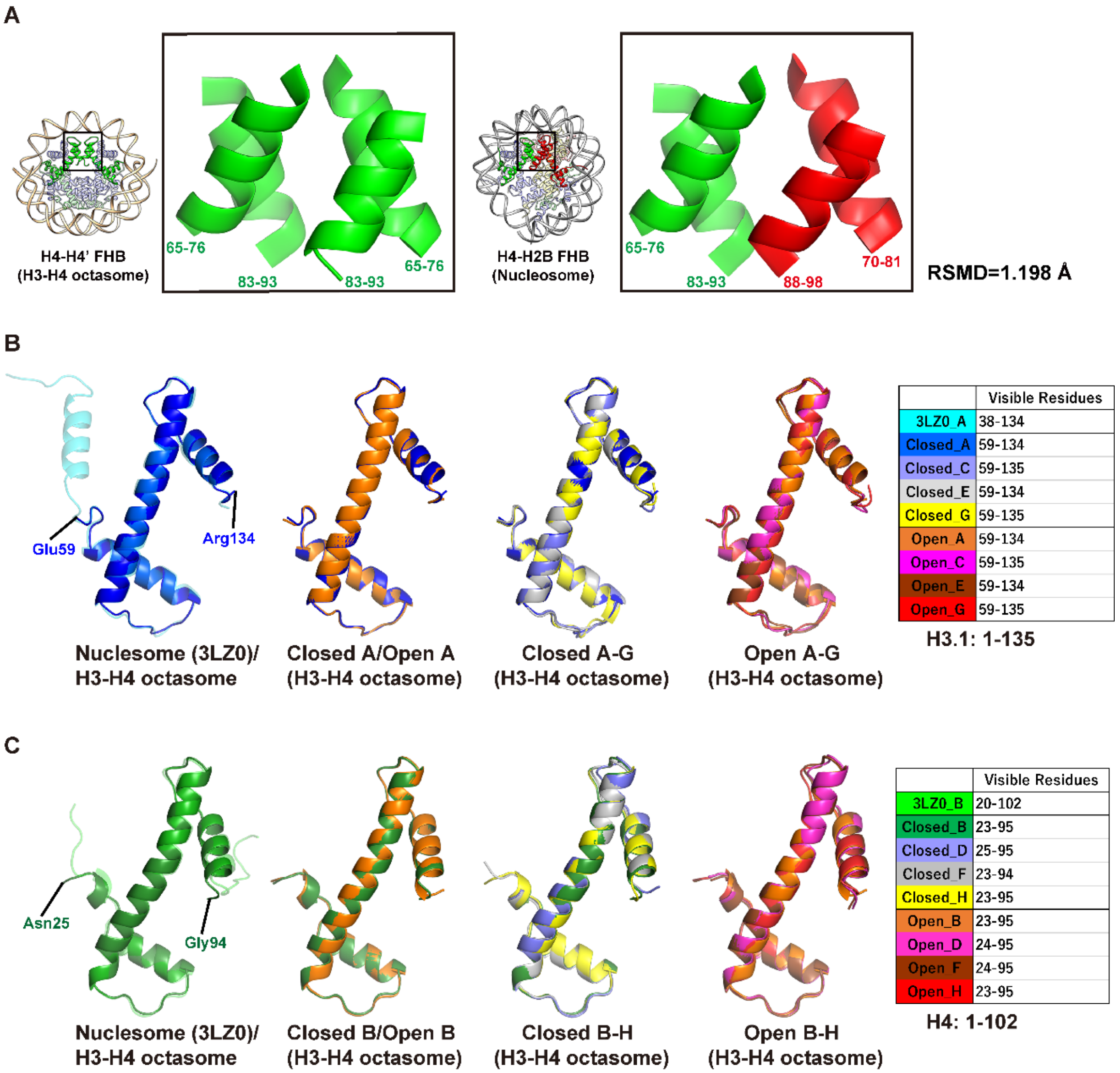

Figure S4. Structural comparison of histones in H3-H4 octasome and nucleosome

(A) Structural comparison of the H4-H4' FHB in the H3-H4 octasome (left panel) and the H4-H2B FHB in the nucleosome (right panel). These FHB structures are superimposable with a root mean square deviation (RMSD) of 1.198 Å for the backbone atoms. (B) Structural comparison of H3.1 in the H3-H4 octasome and the nucleosome. (C) Structural comparison of H4 in the H3-H4 octasome and the nucleosome.

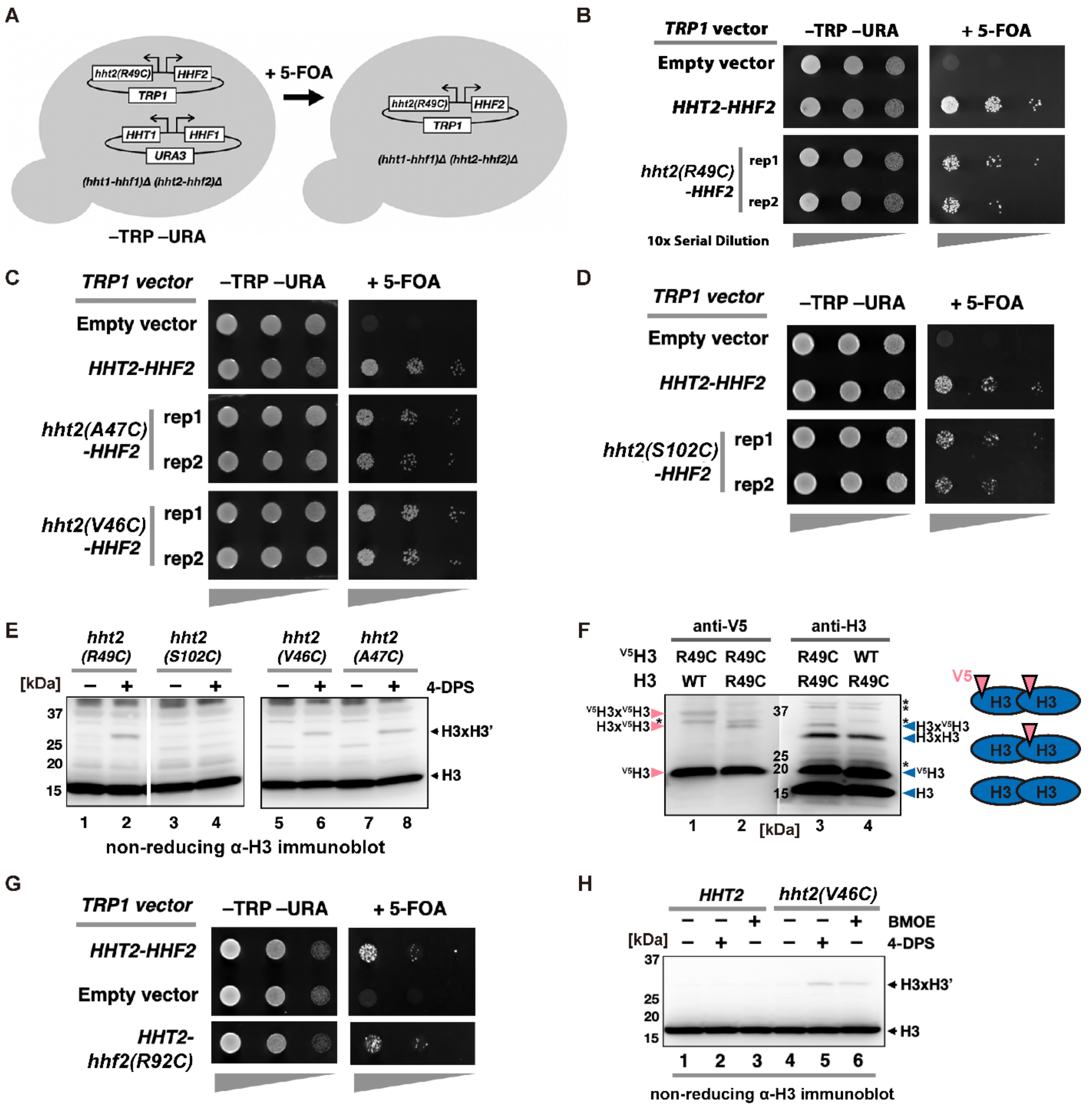

Figure S5. Genetic and biochemical analyses of the H3-H4 octasome containing site-specific cysteine substitutions

(A) The strategy used to verify the functionality of the *hht2(R49C)* gene (2). Plate tests showing the growth of yeast expressing *hht2(R49C)* (B), *hht2(A47C), hht2(V46C)* (C) or *hht2(S102C)* (D) as the sole source of H3. The cells were grown at 30°C under anaerobic conditions, to alleviate the growth defect of *HHT2* mutants grown with 5-FOA. (E) *In vivo* crosslinking analysis of the indicated *HHT2* mutants, performed as described in Figure 4C. The images in lanes 1-4 and lanes 5-8 are taken from two separate immunoblots. (F) *In vivo* crosslinking analysis of V5-tagged H3 (*HHT1*) and untagged H3 (*HHT2*) with R49C or without (WT), performed as described in Figure 4C. However, after the proteins were transferred to a PVDF membrane, the membrane was cut along the molecular weight marker lane (between

samples 2 and 3). The left half of the membrane was probed with an anti-V5 antibody, and the right half with an affinity-purified anti-H3 antibody. The V5 and H3 western images were re-aligned using the marker lane. Asterisks indicate non-specific bands. Blue arrowheads highlight the H3 species detected by anti-H3. Pink arrowheads highlight the V5-containing species. Note that *HHT2* is expressed at a higher level than *HHT1.* As a result, the H3-H3' crosslinked species is biased against the ^{V5}H3(Hht1)-linked species on the anti-H3 immunoblot. The ^{V5}H3x^{V5}H3 adduct, indicated in the anti-V5 blot, is almost undetectable in the anti-H3 western. (G) Plate tests showing the growth of yeast expressing *hhf2(R92C)* as the sole source of H4. (H) *In vivo* crosslinking using 4-DPS and BMOE. WT or *hht2(V46C)* cells were treated with 4-DPS, BMOE, or nothing (control). Protein extracts were analyzed by non-reducing anti-H3 immunoblotting, as in Figure 4C.

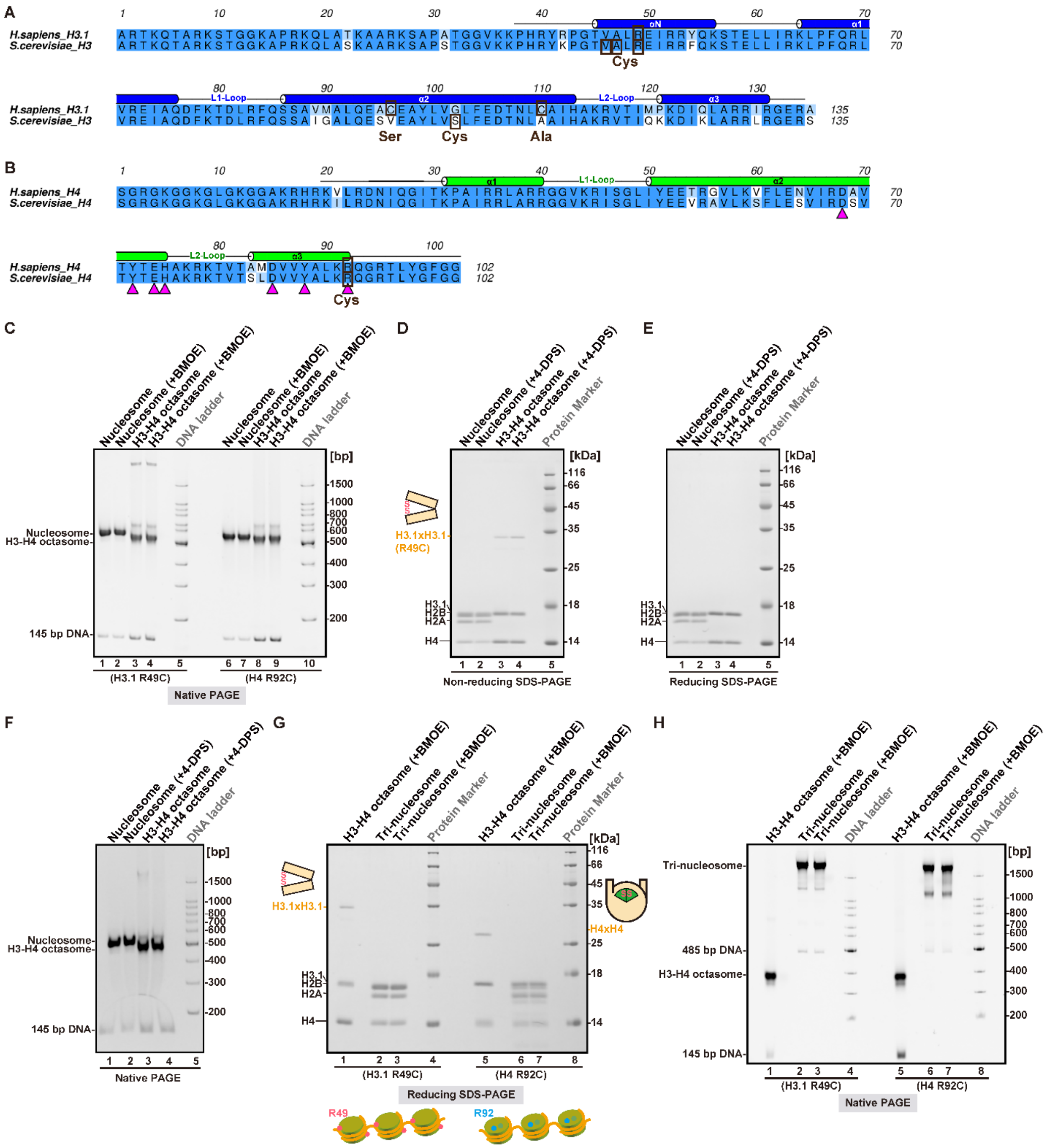

Figure S6. Biochemical analyses of the H3-H4 octasome containing site-specific cysteine substitutions

(A) Sequence alignment of human H3.1 and yeast H3 proteins. The H3.1 mutations used for *in vivo* and *in vitro* crosslinking experiments are indicated with open rectangles. (B) Sequence alignment of human and yeast H4 proteins. The H4 mutations used for *in vivo* and *in vitro* crosslinking experiments are indicated with open rectangles. The amino acids involved in the H4-H4’ FHB formation (pink triangles) are conserved from yeast to humans. (C) Native PAGE of the reconstituted nucleosomes and H3-H4 octasomes, before and after BMOE treatment. Lanes 1-4 contain nucleosomes and H3-H4 octasomes with H3.1 (R49C, C96S,

C110A). Lanes 6-9 contain nucleosomes and H3-H4 octasomes with H4 R92C and H3.1 (C96S, C110A). The DNA was stained with ethidium bromide (EtBr). Reconstituted nucleosomes and H3-H4 octasomes containing the mutant H3.1 (R49C, C96S, C110A) were treated with 4-DPS and subjected to non-reducing (D) and reducing (E) SDS-PAGE. The proteins were stained with Coomassie Brilliant Blue (CBB). (F) Native PAGE (6% polyacrylamide) of the reconstituted nucleosomes and H3-H4 octasomes containing the mutant H3.1 (R49C, C96S, C110A), before and after 4-DPS treatment. The DNA was stained with EtBr. (G) BMOE crosslinking analysis of reconstituted tri-nucleosomes containing human histone H3.1 R49C (which also contains the C96S, C110A substitutions) (lanes 2-3) or H4 R92C and H3.1 (C96S, C110A) (lanes 6-7). Lanes 1 and 5 are H3-H4 octasomes containing respective histone mutants as positive-controls. The nucleoproteins were treated with BMOE and subjected to reducing SDS-PAGE, with CBB staining of the proteins. (H) Native PAGE of the reconstituted tri-nucleosomes, before and after BMOE treatment. Lanes 2-3 contain tri-nucleosomes with H3.1 (R49C, C96S, C110A). Lanes 6-7 contain tri-nucleosomes with H4 R92C and H3.1 (C96S, C110A). Lanes 1 and 5 are H3-H4 octasomes containing the respective histone mutants, after BMOE treatment. The DNA was stained with EtBr.

Table S1: Cryo-EM processing statistics

Sample (H3-H4 octasome)	Closed form (EMD-33010) (PDB: 7X57)	Open form (EMD-33011) (PDB: 7X58)	Intermediate form (EMD-33991) (PDB: 7YOZ)
**Data collection**			
Electron microscope	Krios G3i	Krios G3i	Krios G3i
Camera	K3	K3	K3
Pixel size (Å/pix)	1.05	1.05	1.05
Defocus range (µm)	-1.25 to -2.5	-1.25 to -2.5	-1.25 to -2.5
Exposure time (second)	6	6	6
Total dose (e/Å^2)	63	63	63
Movie frames (no.)	40	40	40
Total micrographs (no.)	5,517	5,517	5,517
**Reconstruction**			
Software	Relion 3.0	Relion 3.0	Relion 3.0
Particles for 2D classification	1,427,558	1,427,558	1,427,558
Particles for 3D classification	990,623	990,623	990,623
Particles in the final map (no.)	186,253	177,542	104,987
Symmetry	C2	C2	C2
Final resolution (Å)	3.6	3.9	4.3
FSC threshold	0.143	0.143	0.143
Map sharpening B factor (Å^2)	-37.53	-35.87	-166.92
**Model building**			
Software	Coot	Coot	Coot
**Refinement**			
Software	Phenix	Phenix	Phenix
**Model composition**			
Protein	980	980	980
Nucleotide	290	290	290
**Validation**			
MolProbity score	1.52	1.39	1.49
Clash score	9.9	7.11	9.15
R.m.s. deviations			
Bond lengths (Å)	0.004	0.003	0.003
Bond angles (˚)	0.783	0.551	0.538
**Ramachandran plot**			
Favored (%)	98.62	99.14	98.62
Allowed (%)	1.38	0.86	1.38
Outliers (%)	0	0	0

Table S2: Yeast Strains

Strain	Genotype	Source
**YYY67**	MAT**a** leu2Δ1 his3Δ200 ura3-52 trp1Δ63 lys2-128δ (hht1-hhf1)Δ::LEU2 (hht2-hhf2)Δ::HIS3 Ty912Δ35-lacZ::his4 <pMS329 (URA3 SUP11 CEN4 ARS4 HHT1-HHF1)>	(3)
**yEL690**	MAT**a** leu2Δ1 his3Δ200 ura3-52 trp1Δ63 lys2-128δ (hht1-hhf1)Δ::LEU2 (hht2-hhf2)Δ::HIS3 Ty912Δ35-lacZ::his4 <pMS329 (URA3 SUP11 CEN4 ARS4 HHT1-HHF1)> <pRS414 (TRP1 CEN6 ARS4)>	This study
**yEL691**	MAT**a** leu2Δ1 his3Δ200 ura3-52 trp1Δ63 lys2-128δ (hht1-hhf1)Δ::LEU2 (hht2-hhf2)Δ::HIS3 Ty912Δ35-lacZ::his4 <pMS329 (URA3 SUP11 CEN4 ARS4 HHT1-HHF1)> <pWZ414-F12 (TRP1 CEN6 ARS4 HHT2-HHF2)>	This study
**yEL698**	MAT**a** leu2Δ1 his3Δ200 ura3-52 trp1Δ63 lys2-128δ (hht1-hhf1)Δ::LEU2 (hht2-hhf2)Δ::HIS3 Ty912Δ35-lacZ::his4 <pMS329 (URA3 SUP11 CEN4 ARS4 HHT1-HHF1)> <pEL629 (TRP1 CEN6 ARS4 hht2(R49C)-HHF2)>	This study
**yEL699**	MAT**a** leu2Δ1 his3Δ200 ura3-52 trp1Δ63 lys2-128δ (hht1-hhf1)Δ::LEU2 (hht2-hhf2)Δ::HIS3 Ty912Δ35-lacZ::his4 <pWZ414-F12 (TRP1 CEN6 ARS4 HHT2-HHF2)>	This study
**yEL703**	MAT**a** leu2Δ1 his3Δ200 ura3-52 trp1Δ63 lys2-128δ (hht1-hhf1)Δ::LEU2 (hht2-hhf2)Δ::HIS3 Ty912Δ35-lacZ::his4 <pEL626 (TRP1 CEN6 ARS4 HHT2-hhf2(R92C))>	This study
**yEL705**	MAT**a** leu2Δ1 his3Δ200 ura3-52 trp1Δ63 lys2-128δ (hht1-hhf1)Δ::LEU2 (hht2-hhf2)Δ::HIS3 Ty912Δ35-lacZ::his4 <pEL629 (TRP1 CEN6 ARS4 hht2(R49C)-HHF2)>	This study
**yEL723**	MAT**a** leu2Δ1 his3Δ200 ura3-52 trp1Δ63 lys2-128δ (hht1-hhf1)Δ::LEU2 (hht2-hhf2)Δ::HIS3 Ty912Δ35-lacZ::his4 <pEL651 (TRP1 CEN6 ARS4 hht2(V46C)-HHF2)>	This study
**yEL724**	MAT**a** leu2Δ1 his3Δ200 ura3-52 trp1Δ63 lys2-128δ (hht1-hhf1)Δ::LEU2 (hht2-hhf2)Δ::HIS3 Ty912Δ35-lacZ::his4 <pEL652 (TRP1 CEN6 ARS4 hht2(A47C)-HHF2)>	This study
**yEL728**	MAT a leu2Δ1 his3Δ200 ura3-52 trp1Δ63 lys2-128δ (hht1-hhf1)Δ::LEU2 (hht2-hhf2)Δ::HIS3 Ty912Δ35-lacZ::his4 <pEL650 (URA3 SUP11 CEN4 ARS4 2xV5-hht1(R49C)-HHF1)> <pWZ414-F12 (TRP1 CEN6 ARS4 HHT2-HHF2)>	This study
**yEL729**	MAT a leu2Δ1 his3Δ200 ura3-52 trp1Δ63 lys2-128δ (hht1-hhf1)Δ::LEU2 (hht2-hhf2)Δ::HIS3 Ty912Δ35-lacZ::his4 <pEL656 (URA3 SUP11 CEN4 ARS4 2xV5-HHT1-HHF1)> <pEL629 (TRP1 CEN6 ARS4 hht2(R49C)-HHF2)>	This study
**yEL730**	MAT a leu2Δ1 his3Δ200 ura3-52 trp1Δ63 lys2-128δ (hht1-hhf1)Δ::LEU2 (hht2-hhf2)Δ::HIS3 Ty912Δ35-lacZ::his4 <pEL650 (URA3 SUP11 CEN4 ARS4 2xV5-hht1(R49C)-HHF1> <pEL629 (TRP1 CEN6 ARS4 hht2(R49C)-HHF2)>	This study
**yEL738**	MAT**a** leu2Δ1 his3Δ200 ura3-52 trp1Δ63 lys2-128δ (hht1-hhf1)Δ::LEU2 (hht2-hhf2)Δ::HIS3 Ty912Δ35-lacZ::his4 <pEL659 (TRP1 CEN6 ARS4 hht2(S102C)-HHF2)>	This study

Table S3: Yeast Plasmids

Plasmid	***Description***	***Source***
pMS329	*URA3 SUP11 CEN4 HHT1-HHF1*	(2)
pRS414	*TRP1 CEN6 ARS4*	(4)
pWZ414-F12	*TRP1 CEN6 ARS4 HHT2-HHF2*	(5)
pEL626	*TRP1 CEN6 ARS4 HHT2-hhf2(R92C)*	This study
pEL629	*TRP1 CEN6 ARS4 hht2(R49C)-HHF2*	This study
pEL649	*URA3 SUP11 CEN4 hht1(R49C)-HHF1*	This study
pEL650	*URA3 SUP11 CEN4 2xV5-hht1(R49C)-HHF1*	This study
pEL651	*TRP1 CEN6 ARS4 hht2(V46C)-HHF2*	This study
pEL652	*TRP1 CEN6 ARS4 hht2(A47C)-HHF2*	This study
pEL656	*URA3 SUP11 CEN4 2xV5-HHT1-HHF1*	This study
pEL659	*TRP1 CEN6 ARS4 hht2(S102C)-HHF2*	This study

References

1. Y. Zi Tan, *et al.*, Addressing preferred specimen orientation in single-particle cryo-EM through tilting. *Nat. Methods 2017 148* **14**, 793–796 (2017).
2. P. Megee, B. Morgan, B. Mittman, M. Smith, Genetic analysis of histone H4: essential role of lysines subject to reversible acetylation. *Science* **247**, 841–845 (1990).
3. Y. Yu, *et al.*, A conserved patch near the C terminus of histone H4 is required for genome stability in budding yeast. *Molecular and Cellular Biology* **31**, 2311–2325 (2011).
4. R. S. Sikorski, P. Hieter, A system of shuttle vectors and yeast host strains designed for efficient manipulation of DNA in Saccharomyces cerevisiae. *Genetics* **122**, 19–27 (1989).
5. W. Zhang, Essential and redundant functions of histone acetylation revealed by mutation of target lysines and loss of the Gcn5p acetyltransferase. *The EMBO Journal* **17**, 3155–3167 (1998).
